# Causes of synthetic disease-modifying drug discontinuation in rheumatoid arthritis: Data from a large real-life cohort

**DOI:** 10.1371/journal.pone.0213219

**Published:** 2019-03-01

**Authors:** Ana Paula Monteiro Gomides, Cleandro Pires de Albuquerque, Ana Beatriz Vargas Santos, Rodrigo Balbino Chaves Amorim, Manoel Barros Bértolo, Paulo Louzada Júnior, Isabela Araújo Santos, Rina Dalva Neubarth Giorgi, Nathalia de Carvalho Sacilotto, Sebastião Cezar Radominski, Fernanda Maria Borghi, Maria Fernanda B. Resende Guimarães, Maria Raquel da Costa Pinto, Gustavo Gomes Resende, Karina Rossi Bonfiglioli, Henrique Carriço da Silva, Maria de Fátima Lobato da Cunha Sauma, Marcel Lobato Sauma, Júlia Brito de Medeiros, Ivânio Alves Pereira, Gláucio Ricardo Wernwer de Castro, Claiton Viegas Brenol, Ricardo Machado Xavier, Licia Maria Henrique da Mota, Geraldo da Rocha Castelar Pinheiro

**Affiliations:** 1 Departamento de Reumatologia, Universidade de Brasília, Brasília, Distrito Federal, Brazil; 2 Departamento do curso de Medicina, Centro Universitário de Brasília- Uniceub, Brasília, Distrito Federal, Brazil; 3 Departamento de Reumatologia, Universidade do Estado do Rio de Janeiro, Rio de Janeiro, Rio de Janeiro, Brazil; 4 Departamento de Reumatologia, Universidade Estadual de Campinas, Campinas, São Paulo, Brazil; 5 Departamento de Reumatologia, Faculdade de Medicina da Universidade de São Paulo, Ribeirão Preto, São Paulo, Brazil; 6 Departamento de Reumatologia, Instituto de Assistência Médica ao Servidor Público Estadual, Hospital do Servidor Público Estadual de São Paulo, São Paulo, São Paulo, Brazil; 7 Departamento de Reumatologia, Universidade Federal do Paraná, Curitiba, Paraná, Brazil; 8 Departamento de Reumatologia, Universidade Estadual de Maringá, Maringá, Paraná, Brazil; 9 Departamento de Reumatologia, Universidade Federal de Minas Gerais, Belo Horizonte, Minas Gerais, Brazil; 10 Departamento de Reumatologia, Universidade de São Paulo, São Paulo, São Paulo, Brazil; 11 Departamento de Reumatologia, Universidade Federal do Pará, Belém, Pará, Brazil; 12 Departamento de Reumatologia, Universidade Federal de Santa Catarina, Florianópolis, Santa Catarina, Brazil; 13 Departamento de Reumatologia, Universidade do Sul de Santa Catarina- Unisul, Florianópolis, Santa Catarina,Brazil; 14 Departamento de Reumatologia, Universidade Federal do Rio Grande do Sul, Porto Alegre, Rio Grande do Sul, Brazil; SERGAS and IDIS, SPAIN

## Abstract

The treatment of rheumatoid arthritis (RA) has evolved rapidly in recent years. Nonetheless, conventional synthetic disease-modifying drugs (csDMARDs) remain the gold standard for RA treatment.

The treatment for RA is expensive and this has a negative impact on public health. Given the low cost of csDMARDs compared to those of other treatment strategies, it is important to manage this type of treatment properly. Information on the duration of use of each drug and the reasons for their discontinuation is relevant to medical practitioners as it could improve the information available regarding side effects and their proper management. Moreover, data from clinical practice in the population can provide health care managers with information for resource allocation and optimization of csDMARD use with a consequent cost reduction in the treatment of RA.

In this cross-sectional study, we aimed to describe the use of csDMARDs in public health services in Brazil, emphasizing on the duration of use and reasons for discontinuation of each drug. This study is a part of the REAL, a multicenter project that evaluated Brazilian patients with RA from eleven rheumatology services from August to October 2015. Patients were examined clinically, and an analysis of complementary exams and medical records was performed.

A total of 1125 patients were included. 98.5% were women with a median age of 55.6 years. 36% and 90.84% patients were using biological disease-modifying drugs (bDMARDs) and csDMARDs, respectively. The duration of use and doses of each medication and the causes of suspension were analyzed.

Most of the patients analyzed in this study were using csDMARDs for prolonged periods and methotrexate showed the longest duration of use. Interruption indexes due to ineffectiveness and side effects were analyzed. The knowledge of common adverse effects may alert attending physicians to the proper management of effective and low-cost therapeutic groups.

## Introduction

The treatment of rheumatoid arthritis (RA) has evolved rapidly. A better understanding of the etiopathogenesis and pathophysiology of this disease allows for the development of drugs targeting new pathways as well as novel therapeutic strategies [1]. Despite the several therapeutic classes, conventional synthetic disease-modifying drugs (csDMARDs) remain the gold standard, either as monotherapy or in combination with biological disease-modifying drugs (bDMARDs) and synthetic target-specific disease-modifying drugs (tsDMARDs) [2] [3]. In addition to their use during treatment, rheumatology societies worldwide recommend the initiation of csDMARDs treatment for naive patients [4] [5]. In its last guidelines for RA treatment, the Brazilian Society of Rheumatology (SBR) suggested the use of csDMARDs in the first line of treatment in up to two different regimens. [3]

The cost of treatment for RA is extremely high and has a negative impact on public health. Brazil is the largest country in Latin America [6] and treatment with bDMARDs is funded by the government, which has been causing huge public spending with a significant economic impact because it is a developing country. RA has become one of the most prevalent public health diseases in proportion to the number of patients. [7] Given the low cost of csDMARDs compared to other treatment strategies, it is extremely important to manage this type of treatment properly.

Knowledge of how this therapeutic class has been used in clinical practice, especially in Brazil, is scarce. Information on the duration of use of each drug as well as the reasons for the discontinuation of these drugs in patients can provide important information for medical practitioners which could in turn, for example, improve the information available to patients regarding side effects and their proper management. In addition, data from clinical practice in this population can provide health care managers with data for resource allocation and optimization of the use of csDMARDs with a consequent cost reduction in the treatment of RA.

The objective of this study was to describe the use of csDMARDs in public health services in Brazil, emphasizing on the duration of use and reasons for discontinuation of each drug.

## Materials & methods

This study is part of the REAL study (Rheumatoid Arthritis in Real Life), a multicenter project that evaluated Brazilian patients with RA [8].

A cross-sectional analysis was performed from August to October 2015. Eleven rheumatology services from different states participated in care provided by the public network. The inclusion criteria were as follows: patients over 18 years, who were diagnosed with RA based on the American Rheumatism Association (ARA) 1987 or the American College of Rheumatology (ACR)/European League Against Rheumatism (EULAR) 2010 classification criteria, and underwent regular monitoring. The patients were examined clinically, and an analysis of complementary exams and medical records was performed. This study was approved by the National Commission of Ethics in Research (CONEP—National Commission of Ethics in Research)—Ministry of Health. All the participants signed in person the written informed consent form.

For statistical analysis of this study, descriptive statistics measures were used, such as frequency measurements and central tendency measures (mean, median) using software SAS 9.4. [9]

## Results

A total of 1125 patients were included in the study, most of whom were women (89.5%), with a median age of 55.6 years. 58.7% belonged to class C and 56.7% were white. The characteristics of the disease can be seen in Table 1.

**Table 1:** Clinical characteristics of the patients with RA in the REAL study [8].

CLINICAL CHARACTERISTICS	ABSOLUTE VALUE OR (%)	n
Disease duration, months, median (min-max)	152.5 (8–683)	1124
Positive rheumatoid factor (%)	78.73	1105
Positive anti-citrulinated peptide antibody	77.2	477
Erosive disease (%)	55.20	1105
Extra articular manifestation	23.3	1115
HAQ, median (min-max)	0.875 (0–3)	1121
CDAI, median (min-max)	9 (0–70)	1122
DAS28 (ESR), median (min-max)	3.52 (0.3–8.24)	932
Time from symtoms to diagnosis, months, median (range)	12 (1–457)	1078

Regarding treatment, we found that 36% and 90.84% were using bDMARDs and csDMARDs, respectively. The distribution of csDMARDs was as follows: 748 patients (66.49%) were treated with methotrexate (MTX), 381 (33.87%) with leflunomide, 120 (10.67%) with hydroxychloroquine, 55 (4.89%) with sulfasalazine, and 26 (2.31%) with chloroquine diphosphate. The most used treatment regimens were MTX + leflunomide (93 patients—8.3%) and MTX + leflunomide + corticoids (49 patients—4.36%).

To meet the study objectives, the duration of csDMARD use and the number of times the treatments were suspended, along with the respective reasons for such suspensions, were analyzed. The duration of use and the doses of each medication are presented in Table 2 and medical causes of suspension of the medications are presented in Table 3.

**Table 2:** Time (in years) of use and doses of synthetic DMARDs in patients with rheumatoid arthritis.

DRUG	MEAN	MEDIAN	MAXIMUM	DOSE (mg) (MEAN/MEDIAN)
CHLOROQUINE DIPHOSPHATE	4.84	3.0	26.0	
HYDROXYCHLOROQUINE	3.26	2.0	19.0	393,33/400
LEFLUNOMIDE	2.63	2.0	16.0	19.79/20
METHOTREXATE	5.74	4.0	30.0	17.35/15.0
SULFASALAZINE	2.27	1.0	12.0	1154.55/1000.00

**Table 3:** Medical causes of interruption of synthetic DMARDs (N/%).

DRUG	PRIMARY INEFFICACY	SECONDARY INEFFICACY	ADVERSE LABORATORIAL EFFECTS	ADVERSE CLINICAL EFFECTS	NUMBER OF ASSESSED CASES OF SUSPENSION
CHLOROQUINE DIPHOSPHATE	66 (31.1)	56 (26.4)	8 (3.8)	82 (38.7)	212
HYDROXYCHLOROQUINE	59 (43)	20 (14.6)	5 (3.6)	53 (38.7)	137
LEFLUNOMIDE	90 (31.6)	81 (28.4)	32 (11.2)	82 (28.8)	285
METHOTREXATE	58 (20.5)	36 (12.7)	58 (20.5)	131 (46.3)	283
SULFASALAZINE	60 (4.5)	38 (28.4)	4 (3)	32 (23.9)	134
TOTAL	333 (31.7)	231(22)	107 (10.2)	380 (36.1)	1051

## Discussion

RA treatment has been increasing in complexity because of multiple therapeutic options. Among all classes, csDMARDs remain the first choice and should be used as early as possible after diagnosis [4] [5]. In a study that analyzed 12-year data from a representative US sample found that among patients with RA, only 47% were using csDMARDs [10].

In a recent publication, Kern et al. [11] pointed out that despite well-established recommendations, there is a gap in the treatment of RA, with a significant percentage of patients not using csDMARDs at the beginning of treatment. In this study, which had a large number of patients (63,101), the authors found that only 51.5% of the patients received csDMARDs as first line of treatment. This may suggest a discrepancy between the scientific recommendations and real-life data.

In our study, we found different data from those reported previously; 90.84% ​​of patients in our study were using csDMARDs. In another study in 14 Latin American countries, it was found that 75% of patients were using this class of therapeutic treatments [12].

Regarding the treatment duration, we found that csDMARDs were being used for prolonged periods, which differs from some studies in literature, which emphasize that most patients discontinue these drugs within the first 3 to 5 years due to intolerance or inefficacy [13].

Among csDMARDs, sulfasalazine was drug that was used for the least amount of time. MTX was used for longer periods.

Regarding suspension of the treatment due to a lack of efficacy, we found that highest indices occurred with antimalarials, followed by leflunomide.

In relation to MTX, Kapral et al. [13] performed a primary inefficacy analysis or an analysis of second attempts to reinitiate MTX after a period of using another DMARD and found that MTX was discontinued in 51 of 79 patients in at least one of two courses (74.6%). In this study, the percentage of interruption due to primary and secondary failure was much lower, at 20.5% and 12.7%, respectively.

Regarding the interruption of MTX due to adverse events, a study with 625 patients showed that the treatment was discontinued due to intolerance in 17.3% of the patients and due to a lack of efficacy in 9% [14]. In our study, we had a higher rate of discontinuation due to adverse effects, with 131 cases (46.3%) discontinuing because of clinical adverse effects and 58 cases (20.5%) of because of laboratory side effects.

In a study published in 2016, it was found that 41.3% of patients discontinued the use of antimalarials, a rate similar to that found in this study [15].

Regarding leflunomide, in the SMILE study, researchers found liver abnormalities in 16% of the patients and neutropenia in 2.3% [16]. Another study showed that in 41.6% of the patients [17], leflunomide was discontinued due to adverse effects, which is a higher index than for other DMARDs—a fact that was not observed in our research. It should be noted that in Brazil, unlike most countries, the combination of MTX and leflunomide is widely used without the increased toxicity as observed in this paper.

Regarding sulfasalazine, we found that in 26.8% there was suspension of the drug due to adverse effects, similar to observations in other studies [18].

Some limitations of this study need to be discussed. The first is in relation to the design of the study. This article is a cross-section of the REAL study, thus not allowing cause-and-effect analysis. The causes of suspension analyzed were only those of a medical nature, and issues inherent to patients, such as adherence to the treatment regime, which may suffer interference from the administration route, for instance, were not included. [19] [20] The numbers of treatment suspensions were analyzed individually; however, one patient may have had several episodes of drug discontinuation for various reasons. Therefore, it was not possible to correlate the causes of interruption with the clinical variables of the patients.

Nevertheless, this study represents the first and an important attempt to evaluate the duration of use and the causes of suspension of DMARDs in Brazil and can serve as a basis for future research.

## Conclusion

Therefore, we conclude that the absolute majority of patients analyzed in this real-life study were using csDMARDs for prolonged periods and that MTX showed the longest duration of use. Interruption indexes due to ineffectiveness and side effects of the drugs were analyzed, providing previously unpublished data from a large Brazilian cohort. The real-life evidence remains critical to the improvement of quality and the cost-effectiveness of RA treatment. Future studies utilizing a similar format should be encouraged.
