# Correction: Causes of synthetic disease-modifying drug discontinuation in rheumatoid arthritis: Data from a large real-life cohort

**DOI:** 10.1371/journal.pone.0215198

**Published:** 2019-04-04

**Authors:** Ana Paula Monteiro Gomides, Cleandro Pires de Albuquerque, Ana Beatriz Vargas Santos, Rodrigo Balbino Chaves Amorim, Manoel Barros Bértolo, Paulo Louzada Júnior, Isabela Araújo Santos, Rina Dalva Neubarth Giorgi, Nathalia de Carvalho Sacilotto, Sebastião Cezar Radominski, Fernanda Maria Borghi, Maria Fernanda B. Resende Guimarães, Maria Raquel da Costa Pinto, Gustavo Gomes Resende, Karina Rossi Bonfiglioli, Henrique Carriço da Silva, Maria de Fátima Lobato da Cunha Sauma, Marcel Lobato Sauma, Júlia Brito de Medeiros, Ivânio Alves Pereira, Gláucio Ricardo Werner de Castro, Claiton Viegas Brenol, Ricardo Machado Xavier, Licia Maria Henrique da Mota, Geraldo da Rocha Castelar Pinheiro

The twenty-first author’s name is spelled incorrectly. The correct name is: Gláucio Ricardo Werner de Castro.
